# Association Between Early Amino Acid Intake and Full-Scale IQ at Age 5 Years Among Infants Born at Less Than 30 Weeks’ Gestation

**DOI:** 10.1001/jamanetworkopen.2021.35452

**Published:** 2021-11-30

**Authors:** Jean-Christophe Rozé, Baptiste Morel, Alexandre Lapillonne, Stéphane Marret, Isabelle Guellec, Dominique Darmaun, Nathalie Bednarek, Thomas Moyon, Laetitia Marchand-Martin, Valérie Benhammou, Véronique Pierrat, Cyril Flamant, Géraldine Gascoin, Delphine Mitanchez, Gilles Cambonie, Laurent Storme, Bathélémie Tosello, Valérie Biran, Olivier Claris, Jean-Charles Picaud, Géraldine Favrais, Alain Beuchée, Gauthier Loron, Catherine Gire, Xavier Durrmeyer, Pierre Gressens, Elie Saliba, Pierre-Yves Ancel

**Affiliations:** 1 Department of Neonatal Medicine, Nantes University Hospital, Nantes, France; 2 Epidémiologie Clinique, Centre d’Investigation Clinique, Nantes University Hospital, Institut National de la Santé et de la Recherche Médicale (INSERM), Nantes, France; 3 Unité Mixte de Recherche (UMR) 1280, Physiologie des Adaptations Nutritionnelles, Nantes University, Institut National de la Recherche Agronomique (INRAE), Nantes, France; 4 UMR 1253, iBrain, Tours University, INSERM, Tours, France; 5 Department of Neonatal Medicine, Assistance Publique Hopitaux de Paris, Necker Enfants Malades Hospital, Paris, France; 6 Department of Neonatal Medicine, Rouen University Hospital, Rouen, France; 7 Department of Neonatal Medicine, Assistance Publique Hopitaux de Paris, Trousseau Hospital, Paris, France; 8 EA 3804, Department of Neonatal Medicine, Reims University Hospital, Champagne-Ardennes University, Reims, France; 9 Obstetrical Perinatal and Pediatric Epidemiology Research Team, U1153 Epidemiology and Biostatistics Sorbonne, University of Paris, INSERM, Paris, France; 10 Department of Neonatal Medicine, Jeanne de Flandre Hospital, Lille University Hospital, Lille, France; 11 Department of Neonatal Medicine, Angers University Hospital, Angers, France; 12 Department of Neonatal Medicine, Tours University Hospital, Tours, France; 13 Department of Neonatal Medicine, Montpellier University Hospital, Montpellier, France; 14 Department of Neonatology, Assistance Publique Hopitaux de Marseille, Aix-Marseille Universite, Marseille, France; 15 Department of Neonatology, University of Paris, Robert-Debre Hospital, Assistance Publique Hopitaux de Paris, Paris, France; 16 Department of Neonatology, Hospices Civils de Lyon, Lyon, France; 17 Department of Neonatology, Rennes University Hospital, Rennes, France; 18 Department of Neonatology, Centre Inter-Communal de Créteil, Créteil, France; 19 NeuroDiderot, Robert-Debré Hospital, University of Paris, INSERM, Paris, France; 20 Clinical Investigation Centre P1419, Assistance Publique-Hôpitaux de Paris, Paris, France

## Abstract

**Importance:**

An international expert committee recently revised its recommendations on amino acid intake for very preterm infants, suggesting that more than 3.50 g/kg/d should be administered only to preterm infants in clinical trials. However, the optimal amino acid intake during the first week after birth in these infants is unknown.

**Objective:**

To evaluate the association between early amino acid intake and cognitive outcomes at age 5 years.

**Design, Setting, and Participants:**

Using the EPIPAGE-2 (Epidemiologic Study on Small-for-Gestational-Age Children—Follow-up at Five and a Half Years) cohort, a nationwide prospective population-based cohort study conducted at 63 neonatal intensive care units in France, a propensity score–matched analysis was performed comparing infants born at less than 30 weeks’ gestation who had high amino acid intake (3.51-4.50 g/kg/d) at 7 days after birth with infants who did not. Participants were recruited between April 1 and December 31, 2011, and followed up from September 1, 2016, to December 31, 2017. Full-scale IQ (FSIQ) was assessed at age 5 years. A confirmatory analysis used neonatal intensive care unit preference for high early amino acid intake as an instrumental variable to account for unmeasured confounding. Statistical analysis was performed from January 15 to May 15, 2021.

**Exposures:**

Amino acid intake at 7 days after birth.

**Main Outcomes and Measures:**

The primary outcome was an FSIQ score greater than −1 SD (ie, ≥93 points) at age 5 years. A complementary analysis was performed to explore the association between amino acid intake at day 7 as a continuous variable and FSIQ score at age 5 years. Data from cerebral magnetic resonance imaging at term were available for a subgroup of preterm infants who participated in the EPIRMEX (Cerebral Abnormalities Detected by MRI, Realized at the Age of Term and the Emergence of Executive Functions) ancillary study.

**Results:**

Among 1789 preterm infants (929 boys [51.9%]; mean [SD] gestational age, 27.17 [1.50] weeks) with data available to determine exposure to amino acid intake of 3.51 to 4.50 g/kg/d at 7 days after birth, 938 infants were exposed, and 851 infants were not; 717 infants from each group could be paired. The primary outcome was known in 396 of 646 exposed infants and 379 of 644 nonexposed infants who were alive at age 5 years and was observed more frequently among exposed vs nonexposed infants (243 infants [61.4%] vs 206 infants [54.4%], respectively; odds ratio [OR], 1.33 [95% CI, 1.00-1.71]; absolute risk increase in events [ie, the likelihood of having an FSIQ score >−1 SD at age 5 years] per 100 infants, 7.01 [95% CI, 0.06-13.87]; *P* = .048). In the matched cohort, correlation was found between amino acid intake per 1.00 g/kg/d at day 7 and FSIQ score at age 5 years (n = 775; β = 2.43 per 1-point increase in FSIQ; 95% CI, 0.27-4.59; *P* = .03), white matter area (n = 134; β = 144 per mm^2^; 95% CI, 3-285 per mm^2^; *P* = .045), anisotropy of the corpus callosum (n = 50; β = 0.018; 95% CI, 0.016-0.021; *P* < .001), left superior longitudinal fasciculus (n = 42; β = 0.018; 95% CI, 0.010-0.025; *P* < .001), and right superior longitudinal fasciculus (n = 42; β = 0.014 [95% CI, 0.005-0.024; *P* = .003) based on magnetic resonance imaging at term. Confirmatory and sensitivity analyses confirmed these results. For example, the adjusted OR for the association between the exposure and the primary outcome was 1.30 (95% CI, 1.16-1.46) using the instrumental variable approach among 978 participants in the overall cohort, and the adjusted OR was 1.35 (95% CI, 1.05-1.75) using multiple imputations among 1290 participants in the matched cohort.

**Conclusions and Relevance:**

In this cohort study, high amino acid intake at 7 days after birth was associated with an increased likelihood of an FSIQ score greater than −1 SD at age 5 years. Well-designed randomized studies with long-term follow-up are needed to confirm the benefit of this nutritional approach.

## Key Points

**Question:**

Is early amino acid intake among very preterm infants associated with cognitive performance at age 5 years?

**Findings:**

In this cohort study of 1789 infants born at less than 30 weeks’ gestation, exposure vs nonexposure to amino acid intake between 3.51 and 4.50 g/kg/d at 7 days after birth was significantly and independently associated with a higher likelihood (61% vs 54%, respectively) of surviving with a full-scale IQ score greater than −1 SD at age 5 years.

**Meaning:**

The results of this study suggest that high early amino acid intake among very preterm infants is safe and significantly associated with improved cognitive outcomes at age 5 years.

## Teaser

This cohort study assesses the association between high amino acid intake among very preterm infants at 7 days after birth and cognitive performance at age 5 years.

## Introduction

Very preterm infants (ie, infants born at <32 weeks’ gestation) are at high risk of developing cognitive difficulties.^1^ Although low early nutritional intake seems to play a role in the occurrence of these difficulties,^2^ the optimal early intake of amino acids is not known. Between 2000 and 2005, international expert committees^2,3,4,5^ recommended providing more than 3.50 g/kg/d of protein at the end of the first week after birth to match the net maternal-fetal amino acid transfer during the third trimester of gestation based on net leucine uptake, obligate protein loss, efficiency of protein retention, and required protein accretion.^6^ In 2018, a European expert committee revised these recommendations and suggested that amino acid intake higher than 3.50 g/kg/d should be administered only as part of clinical trials.^7^

The change in recommendation was likely motivated by the results of randomized clinical trials published between these 2 periods. Observational studies, with their limitations, have emphasized the association between amino acid intake during the first week after birth and short-term neurodevelopmental outcomes^8^ or the association between amino acid intake and brain growth,^9^ accelerated white matter maturation at term,^10^ and more robust functional connectivity in school-aged children who were born preterm^11^; however, no firm conclusion can be drawn from the results of randomized clinical trials.^12^ Only a few randomized studies have assessed the impact of early amino acid intake for neurodevelopment,^13,14,15,16,17^ and the benefit of early amino acid intake of 4.00 g/kg/d remains controversial.^7^ Concern has been expressed because high early amino acid intake was associated with a smaller head circumference among infants of both sexes in 1 study,^16^ and a lower mental developmental index was observed among a subgroup of girls who survived without disability in another study.^17^

Although randomized studies have not yielded clear conclusions because of the difficulties in conducting large randomized clinical trials with long-term follow-up, large observational studies can help to assess a complex therapeutic strategy.^18^ The EPIPAGE-2 (Epidemiologic Study on Small-for-Gestational-Age Children—Follow-up at Five and a Half Years),^1^ a nationwide population-based prospective cohort study, provided a rare and distinct opportunity to explore the association between early amino acid intake and outcomes at age 5 years among unselected preterm infants born before 30 weeks’ gestational age and hospitalized in unselected neonatal intensive care units (NICUs). Exploring this issue was possible because of the wide range of variation in practice associated with low adherence to the recommendations available in 2011,^19^ the period during which the EPIPAGE-2 study was conducted.

## Methods

### Study Population

Recruitment took place at birth in all NICUs in France from April 1 to December 31, 2011. Eligible children were those born at 24 to 29 weeks’ gestation, admitted to the NICU, alive at 7 days after birth, and with information available regarding amino acid intake at 7 days after birth. Children were followed up from September 1, 2016, to December 31, 2017. The study was approved by the National Data Protection Authority, the Consultative Committee on the Treatment of Information on Personal Health Data for Research Purposes, and the Committee for the Protection of People Participating in Biomedical Research.^1^ Recruitment and data collection occurred only after families had received information and provided oral informed consent to participate in the study. This study followed the Strengthening the Reporting of Observational Studies in Epidemiology (STROBE) reporting guideline for cohort studies.

### Amino Acid Intake at 7 Days After Birth

Preterm infants were separated into 2 groups, exposed and nonexposed, based on whether they had been prescribed a high amino acid intake (defined as 3.51-4.50 g/kg/d at 7 days after birth), as recommended by the American National Institute of Child Health and Human Development,^3^ the European Society of Paediatric Gastroenterology, Hepatology and Nutrition,^4^ and the European Society for Clinical Nutrition and Metabolism^5^ in 2011. In the EPIPAGE-2 study, information about nutritional intake was recorded at days 3, 7, and 28 and at hospital discharge. All data were prospectively collected during NICU hospitalization.

### Outcomes

The primary outcome was binary and defined as a full-scale IQ (FSIQ) score greater than −1 SD (ie, ≥93 points) at age 5 years compared with a contemporaneous group of term-born children enrolled in the EPIPAGE-2 follow-up study.^1^ Full-scale IQ was assessed using the French version of the Wechsler Preschool and Primary Scale of Intelligence, 4th edition.^20^ The secondary outcome was FSIQ score at age 5 years considered as a continuous variable. A secondary intermediate outcome was the surface area of white and gray matter and fractional anisotropy (a scalar value between 0 and 1 that describes the extent of anisotropy in the diffusion process, with 0 indicating that diffusion is isotropic and unrestricted in all directions and 1 indicating that diffusion is occurring only along 1 axis and is fully restricted in all other directions) measured by magnetic resonance imaging (MRI) performed at term among the subgroup of preterm infants enrolled in the EPIRMEX (Cerebral Abnormalities Detected by MRI, Realized at the Age of Term and the Emergence of Executive Functions) study,^21^ an ancillary study of the EPIPAGE-2 cohort (eMethods 1 in Supplement 1).

### Statistical Analysis

The main analysis included 1789 children with complete data. We analyzed the association between the exposure and the primary outcome using a propensity score approach^22^ to control for observed confounding factors that might have consequences for both group assignment (exposed vs nonexposed) and outcome. The propensity score of each infant was defined as the probability of having an amino acid intake greater than 3.50 g/kg/d based on the infant’s individual observed covariates. The score was estimated using a logistic regression model, with amino acid intake greater than 3.50 g/kg/d as the dependent variable with regard to baseline maternal, infant, and NICU characteristics. Birth weight *z* scores were based on Olsen curves.^23^ The proportion of participants with missing data ranged from 0% to 8.5%, exceeding 4.0% only for data on Apgar score at 5 minutes and maternal educational level. Missing data were treated as a separate category.

The primary analysis was based on propensity score matching. We used a 1:1 matching algorithm without replacement to match exposed and nonexposed newborns based on propensity score within a caliper of 0.2 SD of the logit of the propensity score.^24^ Imbalance after matching was checked. Odds ratios (ORs) were then calculated to quantify the association between high amino acid intake at day 7 and the primary outcome using logistic regression analysis fit by generalized estimating equations to account for paired data.^25^ Confirmatory analyses were performed for the overall cohort after adjusting for gestational age, sex, birth weight *z* score, and maternal educational level, using the inverse probability weights method and accounting for NICU clustering.^26^

We used an instrumental variable approach as a confirmatory analysis of the principal analysis because propensity score cannot remove hidden biases from unmeasured confounders.^27^ We used NICU preference for high amino acid intake at day 7 as a preference-based measure, with estimated random NICU factors categorized into quartiles (eMethods 2 in Supplement 1).^28,29^ Instrumental variable analysis was performed using the 2-stage residual inclusion approach,^30^ with additional adjustment for gestational age, sex, birth weight *z* score, and maternal educational level.

Complementary analyses were performed using FSIQ score as a continuous variable. First, we measured the Spearman correlation between FSIQ score and amino acid intake as a continuous variable in the matched cohort. In the overall cohort, we used general linear equations and adjustment for gestational age, sex, and birth weight *z* score, weighted by the inverse of the propensity score and accounting for NICU clustering. We completed these analyses by evaluating the Spearman correlation between amino acid intake as a continuous variable and white matter area, gray matter area, and mean fractional anisotropy of cerebral white matter tracts. We compared these outcomes among 4 subgroups of amino acid intake (<3.00, 3.01-3.50, 3.51-4.00, and 4.01-4.50 g/kg/d).

Sensitivity analyses were performed using multiple imputation of missing FSIQ scores. Forty multiple imputation data sets built with predictive mean matching using the mice package in R software, version 4.0.2 (R Foundation for Statistical Computing), were analyzed using variables that were included in the propensity score, morbidities observed during neonatal hospitalization, results from the Ages and Stages questionnaire^31^ (which was completed by parents when children were at the corrected age of 2 years), and deficiencies observed at age 5 years.^1^ Each imputed data set was then analyzed, and the resulting estimates were pooled according to the Rubin rules.^32^ We also analyzed the Spearman correlation between amino acid intake at 7 days after birth and FSIQ score at age 5 years among different subpopulations of participants in the matched cohort with complete data, corresponding to potential confounding factors (presence of bronchopulmonary dysplasia, presence of late-onset sepsis, exposure to maternal breast milk, and presence of severe illness in the first week of life [defined by acute kidney failure and/or the receipt of catecholamine treatment during the first week]). Using the Fine generalized estimating equation, we performed a negative control analysis using survival without morbidity at 36 weeks as a negative control outcome to detect uncontrolled confounding via the propensity score approach.^33^ All tests were 2-sided, and *P* < .05 was considered statistically significant. All analyses were performed using R software, version 4.0.2 (R Foundation for Statistical Computing). Data were analyzed from January 15 to May 15, 2021.

## Results

### Study Population

**Figure 1.**
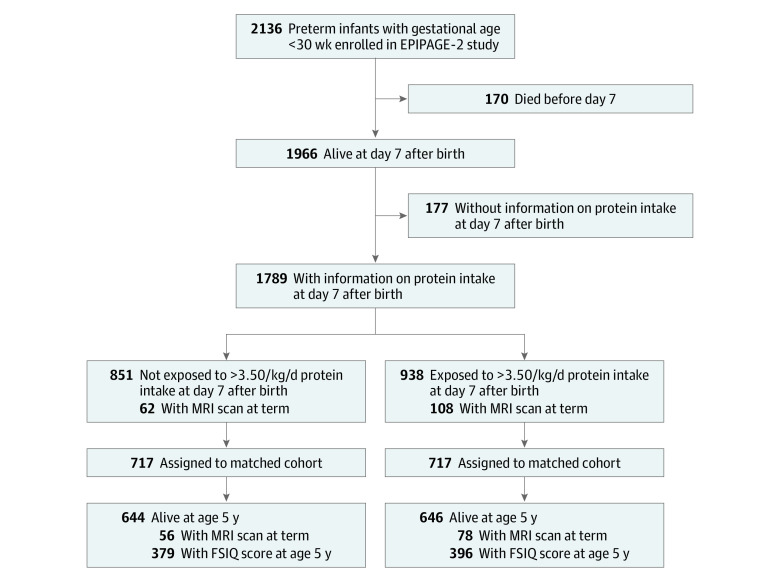
Flowchart of Study Population FSIQ indicates full-scale IQ; MRI, magnetic resonance imaging.

Among 2136 preterm infants admitted to NICUs during the study period, 170 infants died within the first 7 days, leaving 1966 infants alive at day 7. Of those, information about amino acid intake at day 7 was available for 1789 infants (mean [SD] gestational age, 27.17 [1.50] weeks; 929 boys [51.9%]); 860 girls [48.1%]; mean [SD] birth weight *z* score, −0.09 [1.00]); 938 of those infants (52.4%) had amino acid intake greater than 3.50 g/kg/d (exposed group), and 851 infants (47.6%) did not (nonexposed group) (Figure 1). Infants were hospitalized in 63 NICUs across France, and exposure to amino acid intake of 3.51 to 4.50 g/kg/d varied from 0% to 100% (eFigure 1 in Supplement 1). In the overall cohort, infants in the exposed vs nonexposed group were more likely to be female (478 infants [51.0%] vs 382 infants [44.9%], respectively), have mothers with an educational level higher than high school (429 infants [45.7%] vs 316 infants [37.1%]), and have antenatal corticosteroid receipt (615 infants [65.6%] vs 515 infants [60.5%]) but less likely to have acute kidney failure (59 infants [6.3%] vs 107 infants [12.6%]) and assisted ventilation at day 7 (366 infants [39.0%] vs 409 infants [48.1%]). Additional characteristics of exposed vs nonexposed infants in the overall cohort are shown in Table 1.

**Table 1.** Baseline Participant Characteristics

Characteristic	Overall cohort (N = 1789)			Matched cohort (n = 1434)		
	No. (%)		Standardized difference	No. (%)		Standardized difference
	Nonexposed	Exposed		Nonexposed	Exposed	
Total participants, No.	851	938	NA	717	717	NA
Maternal educational level						
<High school	241 (28.3)	231 (24.6)	8.37	194 (27.1)	193 (26.9)	0.32
High school	157 (18.4)	175 (18.7)	0.54	137 (19.1)	134 (18.7)	1.07
>High school	316 (37.1)	429 (45.7)	17.55	291 (40.6)	295 (41.1)	1.12
Missing data	137 (16.1)	103 (11.0)	15.01	95 (13.2)	95 (13.2)	0
Gestational age at birth, mean (SD), wk	27.16 (1.55)	27.18 (1.46)	1.33	27.21 (1.55)	27.17 (1.47)	2.65
Birth weight *z* score, mean (SD)^a^	–0.10 (0.99)	–0.09 (1.01)	1.00	–0.07 (0.97)	–0.07 (1.01)	0
Sex						
Male	469 (55.1)	460 (49.0)	12.11	386 (53.8)	376 (52.4)	2.81
Female	382 (44.9)	478 (51.0)		331 (46.2)	341 (47.6)	
Reason for preterm delivery						
Twin or triplet	288 (33.8)	287 (30.6)	6.94	242 (33.8)	225 (31.4)	5.06
Singleton with preterm labor	239 (28.1)	251 (26.8)	2.96	195 (27.2)	207 (28.9)	3.72
Singleton with preterm rupture of membranes	127 (14.9)	164 (17.5)	6.95	113 (15.8)	114 (15.9)	0.38
Singleton with vascular disorders and FGR	66 (7.8)	71 (7.6)	0.71	55 (7.7)	56 (7.8)	0.52
Singleton with vascular disorders and no FGR	76 (8.9)	82 (8.7)	0.67	63 (8.8)	63 (8.8)	0
Singleton with placental abruption	11 (1.3)	13 (1.4)	0.87	10 (1.4)	10 (1.4)	0
Singleton with isolated FGR	24 (2.8)	34 (3.6)	4.53	22 (3.1)	23 (3.2)	0.80
Missing data	20 (2.4)	36 (3.8)	8.61	17 (2.4)	19 (2.6)	1.79
Antenatal corticosteroid receipt						
No	143 (16.8)	160 (17.1)	0.69	114 (15.9)	122 (17.0)	3.02
Yes	515 (60.5)	615 (65.6)	10.48	457 (63.7)	451 (62.9)	1.72
Incomplete cure	163 (19.2)	130 (13.9)	14.27	116 (16.2)	115 (16.0)	0.38
Missing data	30 (3.5)	33 (3.5)	0.05	30 (4.2)	29 (4.0)	0.71
Cesarean delivery						
Yes	530 (62.3)	563 (60.0)	4.64	450 (62.8)	435 (60.7)	4.30
Missing data	13 (1.5)	8 (0.9)	6.27	5 (0.7)	6 (0.8)	1.60
Apgar score ≥7 at 5 min after birth						
Yes	586 (68.9)	684 (72.9)	8.95	505 (70.4)	507 (70.7)	0.61
Missing data	77 (9.0)	54 (5.8)	12.59	52 (7.3)	50 (7.0)	1.09
Regular intestinal transit during first week after birth						
Yes	430 (50.5)	499 (53.2)	5.35	359 (50.1)	379 (52.9)	5.58
Missing data	34 (4.0)	43 (4.6)	2.86	31 (4.3)	31 (4.3)	0
Acute kidney failure						
Yes	107 (12.6)	59 (6.3)	17.31	59 (8.2)	56 (7.8)	1.55
Missing data	23 (2.7)	31 (3.3)	3.52	20 (2.8)	19 (2.6)	2.54
Surfactant receipt						
No	143 (16.8)	149 (15.9)	2.49	127 (17.7)	117 (16.3)	3.70
1 Dose	493 (57.9)	578 (61.6)	7.53	421 (58.7)	430 (60.0)	2.55
>2 Doses	211 (24.8)	209 (22.3)	5.9	167 (23.3)	168 (23.4)	0.33
Missing data	4 (0.5)	2 (0.2)	4.47	2 (0.3)	2 (0.3)	0
Assisted ventilation at day 7						
Yes	409 (48.1)	366 (39.0)	18.31	314 (43.79)	282 (39.3)	9.06
Missing data	3 (0.4)	11 (1.2)	9.45	2 (0.3)	4 (0.6)	4.33
Volume of NICU in which infant was hospitalized at day 7, No. of infants						
<20	183 (21.5)	113 (12.0)	25.50	124 (17.3)	108 (15.1)	6.06
21-30	150 (17.6)	227 (24.2)	16.21	137 (19.1)	145 (20.2)	2.79
31-40	132 (15.5)	111 (11.8)	10.73	102 (14.2)	104 (14.5)	0.77
>40	386 (45.4)	487 (51.9)	13.15	354 (49.4)	360 (50.2)	1.68

Abbreviations: FGR, fetal growth restriction; NA, not applicable; NICU, neonatal intensive care unit.

^a^ Birth weight *z* score based on Olsen curves.^23^

### Propensity Score–Matched Analysis

Propensity scores were calculated for 1789 infants in the overall cohort and ranged from 0.095 to 0.922. Distributions of propensity scores are summarized in eFigure 2 in Supplement 1. The area under the receiver operating characteristic curve for the propensity score model was 0.67 (95% CI, 0.64-0.69). A total of 1434 of 1789 infants could be matched, with 717 infants in each group (exposed and nonexposed). The matched groups were well balanced in all recorded baseline variables (eg, exposed infants: 341 girls [46.7%] with a mean [SD] gestational age of 27.17 [1.47] weeks; nonexposed infants: 331 girls [46.2%] with a mean [SD] gestational age of 27.21 [1.55] weeks) (Table 1).

Characteristics of nutritional intake at 3, 7, and 28 days after birth and outcomes at 36 weeks’ postmenstrual age are shown in Table 2. Nutritional intakes at day 3 and day 7 were significantly correlated (Spearman *r* = 0.41; *P* < .001) (eFigure 3 in Supplement 1).

**Table 2.** Characteristics and Outcomes of Nonexposed vs Exposed Infants

Characteristic or outcome	Overall cohort (n = 1789)					Matched cohort (n = 1434)				
	Infants, No. (n = 851)	Nonexposed	Infants, No. (n = 938)	Exposed	*P* value	Infants, No. (n = 717)	Nonexposed	Infants, No. (n = 717)	Exposed	*P* value
**Nutritional intake, mean (SD)**										
Day 3										
Total volume, mL/kg/d	841	127.0 (26.0)	928	137.0 (26.0)	NA	710	128.0 (26.0)	712	136 (26.0)	NA
Percentage of parenteral nutrition	837	89.0 (57.0)	924	90 (12.0)		706	88 (54.0)	708	92 (38.0)	
Protein, g/kg/d^a^	830	2.5 (0.8)	928	3.2 (0.7)		704	2.5 (0.8)	712	3.2 (0.7)	
Carbohydrates, g/kg/d	836	10.9 (3.0)	931	11.3 (2.9)		706	10.9 (3.0)	714	11.2 (2.9)	
Lipids, g/kg/d	826	1.6 (1.1)	922	2.0 (1.1)		701	1.7 (1.1)	705	2.0 (1.1)	
Day 7										
Total volume, mL/kg/d	851	151.0 (28.0)	938	164.0 (22.0)	NA	714	152.0 (28.0)	715	165.0 (22.0)	NA
Percentage of parenteral nutrition	851	78.0 (34.0)	938	80.0 (20.0)		694	76.0 (36.0)	705	80.0 (20.0)	
Protein, g/kg/d^a^	851	2.9 (0.6)	938	4.0 (0.2)		717	3.0 (0.5)	717	4.0 (0.2)	
Carbohydrates, g/kg/d	849	13.6 (3.7)	937	15.0 (3.6)		716	13.8 (3.6)	716	14.9 (3.5)	
Lipids, g/kg/d	840	3.0 (1.5)	937	3.6 (1.6)		715	3.1 (1.5)	716	3.6 (1.7)	
Receiving maternal breast milk, No. (%)	851	144 (16.9)	938	122 (13.0)		717	126 (17.6)	717	99 (13.8)	
Day 28										
Alive, No. (%)	851	769 (90.4)	938	887 (94.6)	NA	717	669 (93.3)	717	671 (93.6)	NA
Total volume, mL/kg/d	723	158 (50.0)	820	159.0 (23.0)		651	155.0 (29.0)	642	159.0 (33.0)	
Percentage of parenteral nutrition	723	30 (37.0)	820	26.0 (35.0)		654	26.1 (35.2)	652	25.2 (33.5)	
Protein, g/kg/d^a^	624	3.1 (0.7)	701	3.2 (0.7)		539	3.1 (0.7)	534	3.2 (0.7)	
Carbohydrates, g/kg/d	633	14.6 (3.4)	706	13.9 (3.9)		546	14.6 (3.3)	537	14.3 (3.5)	
Lipids, g/kg/d	627	5.0 (2.0)	701	5.2 (1.9)		541	5.1 (2.0)	533	5.2 (1.8)	
Receiving maternal breast milk, No. (%)	851	154 (18.1)	938	147 (15.7)		717	134 (18.7)	717	114 (15.9)	
**Outcome at 36 wk postmenstrual age**										
Alive, No. (%)	851	746 (87.7)	938	871 (92.9)	NA	717	652 (90.9)	717	657 (91.6)	NA
Alive without severe morbidity, No. (%)^b^	821	528 (64.3)	881	619 (70.3)		652	432 (66.3)	635	440 (69.3)	
Delta weight *z* score between discharge and birth, mean (SD)	692	−1.26 (0.75)	791	−1.11 (0.74)		603	−1.25 (0.74)	590	−1.12 (0.72)	
Duration of any assisted ventilation up to 36 wk, mean (SD), d	843	32.4 (23.5)	932	30.2 (23.7)		644	33.8 (24.0)	655	31.7 (24.0)	
Duration of parenteral nutrition up to 36 wk, mean (SD), d	668	27.3 (15.3)	784	26.0 (12.0)		585	26.6 (15.1)	589	26.1 (12.2)	
**Type of nutrition at discharge, No. (%)**										
Alive at discharge	851	741 (87.1)	938	864 (92.1)	NA	717	649 (90.5)	717	651 (90.8)	NA
Maternal breast milk	741	237 (32.0)	864	307 (35.5)		649	212 (32.7)	651	221 (33.9)	
Nutrient-enriched maternal breast milk	741	112 (15.1)	864	114 (13.2)		649	102 (15.7)	651	83 (12.7)	
Donated breast milk	741	23 (3.1)	864	21 (2.4)		649	16 (2.5)	651	13 (2.0)	
Nutrient-enriched formula	741	183 (24.7)	864	219 (25.3)		649	165 (25.4)	651	176 (27.0)	
Standard formula	741	18 (2.4)	864	16 (1.9)		649	15 (2.3)	651	10 (1.5)	
Specific formula	741	94 (12.7)	864	86 (10.0)		649	76 (11.7)	651	66 (10.1)	
Missing data	741	74 (10.0)	864	74 (8.6)		649	63 (9.7)	651	82 (12.6)	
**Cognitive outcomes at age 5 y**										
Alive at age 5 y, No. (%)	851	735 (86.4)	938	858 (91.5)	.001	717	644 (89.8)	717	646 (90.1)	.86
FSIQ										
Data available, No. (%)	735	432 (58.8)	858	546 (63.6)	.047	644	379 (58.9)	646	396 (61.3)	.37
Score, mean (SD)	432	92.3 (15.7)	546	95.7 (15.6)	.001	379	93.6 (15.2)	396	95.7 (15.5)	.03
Score ≥93, No. (%)^c^	432	205 (47.5)	546	208 (38.1)	.003	379	206 (54.4)	396	243 (61.4)	.048
WPPSI-IV score, mean (SD)										
Verbal comprehension	436	96.0 (17.1)	550	98.4 (17.1)	.03	383	97.0 (16.9)	399	98.8 (16.6)	.30
Visual-perceptual reasoning	439	93.6 (15.5)	551	95.9 (14.7)	.02	385	94.5 (15.0)	401	96.1 (14.4)	.13
Fluid reasoning	435	94.6 (15.8)	551	97.1 (14.8)	.01	384	95.1 (15.4)	402	97.3 (14.6)	.04
Working memory	437	93.0 (15.2)	548	95.1 (13.7)	.02	385	93.6 (13.7)	400	95.2 (14.0)	.09
Processing speed	433	93.7 (15.2)	548	95.2 (15.4)	.12	381	94.3 (14.9)	399	95.0 (15.6)	.52

Abbreviations: FSIQ, full-scale IQ; NA, not applicable; WPPSI-IV, Wechsler Preschool and Primary Scale of Intelligence, 4th edition.

^a^ Sum of enteral protein and intravenous amino acid supply.

^b^ Severe morbidity was defined by the presence of one of the following events: intraventricular hemorrhage with ventricular dilatation (grade 3), intraparenchymal hemorrhage (defined as large unilateral parenchymal hyperdensity or large unilateral porencephalic cyst), cystic periventricular leukomalacia (defined as periventricular white matter echolucencies during ultrasonography), severe bronchodysplasia treatment with oxygen for at least 28 days with need for oxygen at 30% or higher, receipt of mechanical ventilation, continuous positive airway pressure at 36 weeks’ postmenstrual age, necrotizing enterocolitis (Bell stage 2 or 3), or retinopathy at stage 3 or higher.

^c^ Equivalent to *z* score of −1 SD.

**Figure 2.**
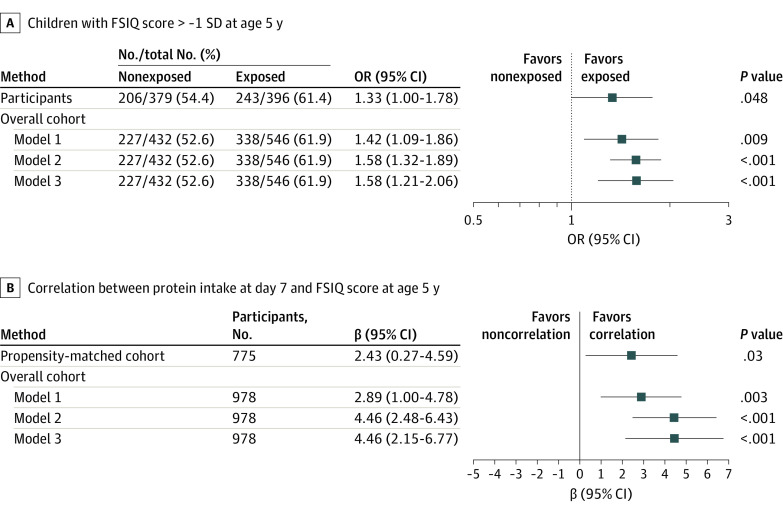
Multivariable Analysis of the Association Between Amino Acid Intake at 7 Days After Birth and FSIQ at Age 5 Years Among infants with complete data available. Model 1 was adjusted for gestational age, sex, birth weight *z* score, and maternal educational level. Model 2 was adjusted for gestational age and weighted by the inverse of the propensity score. Model 3 was adjusted for gestational age and weighted by the inverse of the propensity score, accounting for neonatal intensive care unit clustering. A, The position of each square represents the point estimate of the exposure effect. Horizontal lines represent 95% CIs. Results are expressed as number of events per number of participants. B, The position of each square represents the point estimate of the β coefficient between amino acid intake at 7 days after birth as a continuous variable and FSIQ at age 5 years. FSIQ indicates full-scale IQ; OR, odds ratio.

In the matched cohort, survival rates at age 5 years were similar between the exposed (646 infants [90.1%]) and nonexposed (644 infants [89.8%]) groups. The primary outcome was known for 396 of 646 exposed infants (61.3%) and 379 of 644 nonexposed infants (58.9%) who were alive at age 5 years (Table 2). The characteristics of preterm infants alive at age 5 years with vs without FSIQ scores were not significantly different (eg, 408 of 775 boys [52.6%] vs 275 of 515 boys [53.4%], respectively; mean [SD] birth weight *z* score, −0.09 [0.99] vs −0.02 [0.97]; mean [SD] gestational age, 27.3 [1.5] weeks in both groups) (eTable 1 in Supplement 1). An FSIQ score greater than −1 SD (ie, ≥93 points) was observed more frequently in exposed vs nonexposed infants (243 infants [61.4%] vs 206 infants [54.4%], respectively; OR, 1.33; 95% CI, 1.00-1.78; absolute risk increase in events [ie, the likelihood of having an FSIQ score >−1 SD at age 5 years] per 100 infants, 7.01 [95% CI, 0.06-13.87]; *P* = .048) (Table 2; Figure 2). The ORs for an FSIQ score greater than −1 SD were 0.99 (95% CI, 0.67-1.47) among 408 boys, 1.89 (95% CI, 1.23-2.88) among 367 girls, 1.54 (95% CI, 0.92-2.59) among 237 infants born at 24 to 26 weeks’ gestation, and 1.27 (95% CI, 0.90-1.79) among 538 infants born at 27 to 29 weeks’ gestation.

### Inverse Probability of Treatment Weighted Analysis

In the analysis accounting for NICU clustering, the OR for the association between the exposure and the primary outcome, adjusted for gestational age and weighted by the inverse of the propensity score, was 1.58 (95% CI, 1.21-2.06) among 978 infants in the overall cohort (Figure 2).

### Instrumental Variable Analysis

In the overall cohort, the instrumental variable (ie, NICU preference) was associated with an amino acid intake between 3.51 and 4.50 g/kg/d at day 7 (eg, 8.6% in quartile 1 vs 41.7% in quartile 4 among exposed infants; *P* < .001) (eTable 2 in Supplement 1) but not with antenatal corticosteroid receipt (eg, 58.0% in quartile 1 vs 63.6% in quartile 4; *P* = .08), inborn status (eg, 85.0% in quartile vs 88.4% in quartile 4; *P* = .13), or NICU patient volume (eg, 16.3% in quartile 1 vs 19.5% in quartile 4 for small units with <20 enrolled infants; *P* = .20), which are quality markers that are usually associated with outcomes (eTable 3 in Supplement 1). The instrumental variable was not associated with other practice strategies already assessed in the EPIPAGE-2 study (eg, difference between observed and expected percentage, −8.0% in quartile 1 vs −2.6% in quartile 4 [*P* = .42] for skin contact during the first 7 days; 6.9% in quartile 1 vs 1.2% in quartile 4 [*P* = .37] for sedation during the first 7 days) (eFigure 4 in Supplement 1).^34^ Using this instrumental variable approach, the partial *F* statistic for the instrumental variable in the first-stage model was 139, and the adjusted OR for the association between the exposure and the primary outcome among 978 participants was 1.30 (95% CI, 1.16-1.46) after adjustment for gestational age, birth weight *z* score, sex, and maternal educational level.

### Complementary Analyses

In the matched cohort, among 775 participants, a significant correlation was found between amino acid intake per 1.00 g/kg/d at day 7 and FSIQ score at 5 years (β = 2.43 per 1-point increase in FSIQ score; 95% CI, 0.27-4.59; *P* = .03). All correlations between amino acid intake at day 7 and FSIQ score as a continuous variable are shown in Figure 2. Among all correlated nutritional intake levels, only amino-acid intake levels at days 3 and 7 were correlated with FSIQ score (Spearman *r* = 0.09 and 0.11, respectively) (eFigure 3 in Supplement 1).

Magnetic resonance imaging data were available for 170 infants in the overall cohort and 134 infants in the matched cohort. The gestational ages and birth weight *z* scores were not significantly different between the subgroup of infants with MRI data vs the entire cohort (mean [SD] gestational age, 27.36 [1.33] weeks vs 27.15 [1.35] weeks, respectively; *P* = .14; mean [SD] *z* score, –0.09 [1.00] vs −0.08 [0.96]; *P* = .82). In both the overall and matched cohorts, amino acid intake at day 7 was correlated with white matter area (overall cohort: n = 170; β = 170 [95% CI, 30-310; *P* = .02]; matched cohort: n = 134; β = 144 per mm^2^ [95% CI, 3-285 per mm^2^; *P* = .045]), anisotropy of the corpus callosum (overall cohort: n = 62; β = 0.018 [95% CI, 0.015-0.021; *P* < .001]; matched cohort: n = 50; β = 0.018 [95% CI, 0.016-0.021; *P* < .001]), left superior longitudinal fasciculus (overall cohort: n = 51; β = 0.017 [95% CI, 0.010-0.025; *P* < .001]; matched cohort: n = 42; β = 0.018 [95% CI, 0.010-0.025; *P* < .001]), and right superior longitudinal fasciculus (overall cohort: n = 51; β = 0.012 [95% CI, 0.005-0.019; *P* = .002]; matched cohort: n = 42; β = 0.014 [95% CI, 0.005-0.024; *P* = .003]) measured by MRI at term (eTable 4 and eTable 5 in Supplement 1).

**Figure 3.**
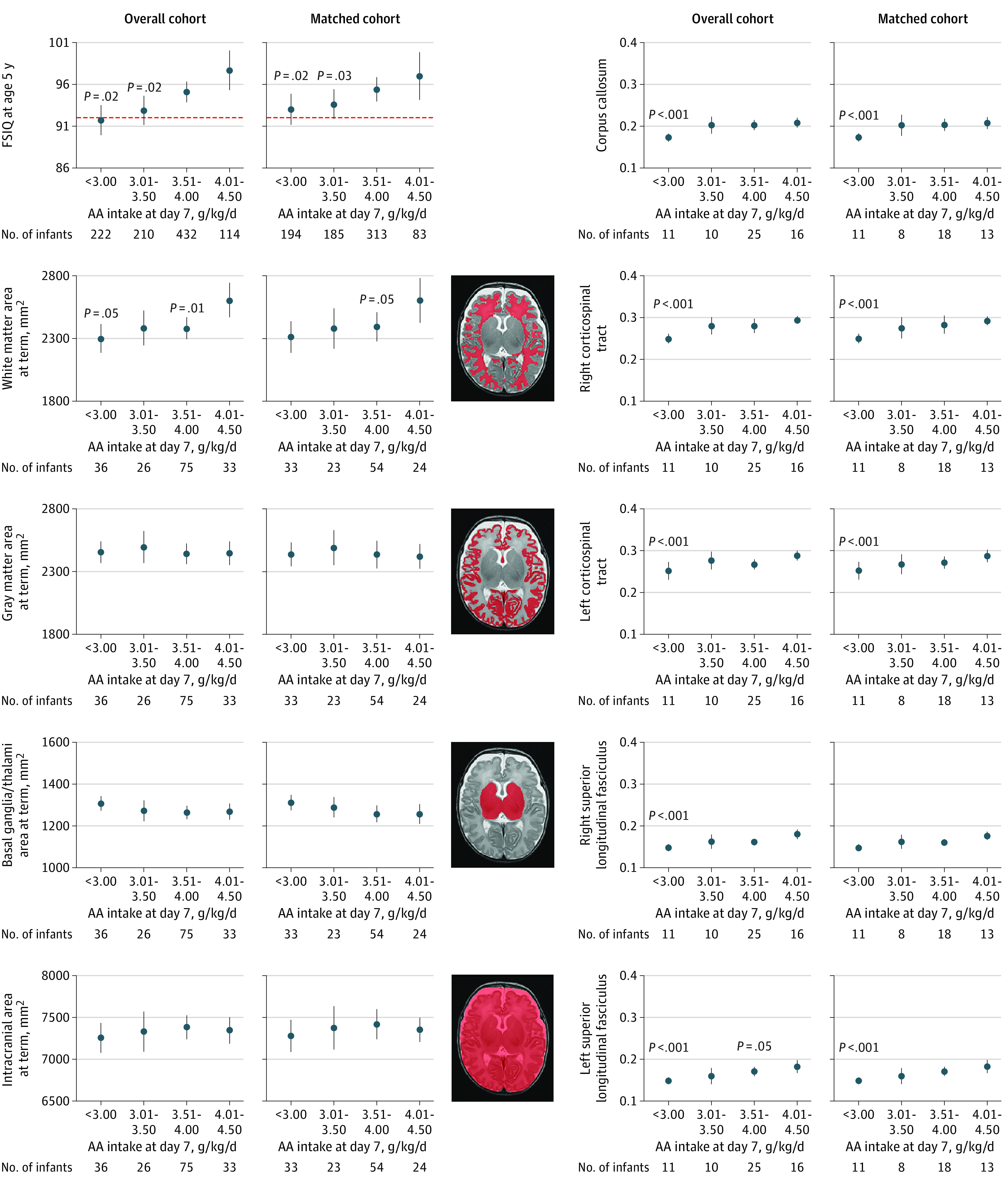
Association Between Amino Acid (AA) Intake at 7 Days After Birth, Full-Scale IQ (FSIQ) at Age 5 Years, and Magnetic Resonance Imaging (MRI) Results at Equivalent Term, Categorized by 4 Subgroups of AA Intake Adjusted for gestational age, biological sex, and birth weight *z* score weighted by the inverse of the propensity score and accounting for neonatal intensive care unit clustering. The dotted red line (graphs on top left) represents −1 SD of FSIQ among full-term infants enrolled in the EPIPAGE-2 follow-up study.^1^ Fractional anisotropy (graphs on right) is a scalar value between 0 and 1 that describes the extent of anisotropy in the diffusion process. A value of 0 indicates that diffusion is isotropic and unrestricted in all directions. A value of 1 indicates that diffusion is occurring only along 1 axis and is fully restricted in all other directions. The reference population is the population of full-term infants enrolled in the EPIPAGE-2 follow-up study.^1^ This reference population was not hospitalized during the neonatal period; therefore, the protein intake on day 7 was unknown and very low because protein was provided solely from milk. The equivalent term is the conventional formulation used to indicate the MRI was performed at approximately 40 weeks’ postmenstrual age (ie, corrected age).

The association between amino acid intake at day 7 (split into 4 subgroups: <3.00, 3.01-3.50, 3.51-4.00, and 4.01-4.50 g/kg/d), FSIQ score, and MRI data are shown in Figure 3. In the matched cohort, the white matter area was significantly smaller among 56 infants with amino acid intake of 3.51 to 4.00 g/kg/d (mean [SD] area, 2380 [500] mm^2^) compared with 78 infants with amino acid intake of 4.01 to 4.50 g/kg/d (mean [SD] area, 2580 [510] mm^2^; *P* = .02) after adjusting for gestational age, birth weight *z* score, and sex and weighting by the inverse of the propensity score. The mean fractional anisotropy of the corpus callosum was significantly smaller among 13 infants with amino acid intake of less than 3.00 g/kg/d (mean [SD], 0.17 [0.02]) compared with 11 infants with intake of 4.01 to 4.50 g/kg/d (mean [SD], 0.21 [0.03]; *P* < .001) after adjustment.

Amino acid intake at 7 days after birth was significantly correlated with carbohydrate intake (Spearman *r* = 0.10; *P* < .001) and lipid intake (Spearman *r* = 0.12; *P* < .001), but neither carbohydrate intake (Spearman *r* = 0.03; *P* = .46) nor lipid intake (Spearman *r* = 0.02; *P* = .66) was correlated with FSIQ score (eTable 6 and eFigure 4 in Supplement 1). In the overall cohort, the negative control analysis of propensity score did not reveal any association between early amino acid intake greater than 3.50 g/kg/d and survival without morbidity at 36 weeks’ postmenstrual age (OR, 1.01; 95% CI, 0.76-1.35) after adjustment for propensity score.

### Sensitivity Analyses

When multiple imputations were used in the matched cohort, the adjusted OR for the association between the exposure and the primary outcome was 1.35 (95% CI, 1.05-1.75). In the overall cohort, the observed correlation between amino acid intake and FSIQ score using multiple imputation based on propensity score matching or weighting by the inverse of propensity score was consistent with the analysis among participants with complete data (eFigure 5 in Supplement 1). The correlations between amino acid intake and FSIQ score among subgroups of the matched cohort, corresponding to potential confounding factors, are shown in eTable 7 in Supplement 1. Among 226 of 465 infants with severe illness during the first week of life, the β correlation was 4.05 (95% CI, 0.98-7.12; *P* = .01), and higher amino acid intake at day 7 was not associated with an increased risk of death (OR, 1.03; 95% CI, 0.62-1.73; *P* = .90).

## Discussion

In the EPIPAGE-2 nationwide population-based cohort study, amino acid intake higher than 3.50 g/kg/d at 7 days after birth, used as a proxy for early amino acid intake, was independently associated with an increase in the likelihood of surviving with an FSIQ score greater than −1 SD at age 5 years. Moreover, we observed a correlation between amino acid intake at 7 days after birth as a continuous variable and FSIQ score. Among a subgroup of infants enrolled in the EPIRMEX study, a correlation was found between amino acid intake and white matter area or anisotropy of several white matter tracts identified by an MRI scan performed at term.

These findings are inconsistent with several randomized studies that did not find any significant benefit during follow-up among children exposed to high early amino acid intake. However, the statistical power of those studies was relatively weak, and the follow-up extended to only 18 or 24 months of corrected age, which were clear limitations. The significant positive association between FSIQ score and amino acid intake observed among girls in the current study was consistent with results from an earlier observational study comparing 2 consecutive cohorts in which preterm female infants with higher amino acid intake had an increased likelihood of reaching a Bayley mental developmental index of 85 points or higher at age 2 years.^35^ This association, which was observed mainly in girls, is probably explained by differences in metabolism and body composition between girls and boys, suggesting the need to perform prespecified sex-specific analyses in future randomized clinical trials.^36^

The association between early nutrition and MRI results has been reported in few studies of preterm infants.^10,11,37,38,39,40^ The designs, findings from MRI, and characteristics of nutritional support varied between these studies and likely accounted for their inconsistent conclusions. Some studies^9,10,11,38^ found an association between nutrition, brain growth, and accelerated white matter maturation, whereas others^37,39^ did not. In our study, early amino acid intake was associated with increases in white matter area and increased anisotropy of several white matter tracts assessed by MRI at term. In a recent study by the Protein, Insulin, and Neonatal Outcomes (PIANO) group,^11^ in which MRI scans were obtained at age 7 years, the investigators noted that greater neonatal amino acid intake was positively associated with connectivity strength. These studies^9,10,11,38^ suggest that optimizing early protein intake in preterm neonates may represent a potential avenue to improve brain maturation in very preterm infants. These findings^10,11,38^ are consistent with an experimental study in rodents,^41^ which reported that protein deficiency during brain development had negative consequences for white matter development and maturation.

### Strengths and Limitations

This study has several strengths. These strengths include the population-based cohort design and the prospective enrollment of all infants born preterm in France in 2011. Furthermore, the data on nutrition were prospectively collected using a detailed standardized questionnaire. Therefore, comprehensive and accurate information on nutritional strategies was available. We observed a significant correlation between amino acid, carbohydrate, and lipid intake at day 7, but only amino acid intake was correlated with FSIQ score at age 5 years. The difference in nutritional strategies observed during the first week of life was no longer observed at day 28 or at discharge, emphasizing the importance of early nutrition in the association with long-term outcomes. Moreover, we did not observe any increase in mortality among exposed preterm infants with initial severe illness, contrary to what was observed among critically ill term infants exposed to early amino acid intake.^42^

This study also has limitations. The main limitation is potential uncontrolled confounding. We used multiple statistical approaches to reduce bias as much as possible. To control for the indication bias inherent in this type of study, we performed a propensity score analysis and rigorously adjusted for confounding factors, minimizing the likelihood of incorrectly attributing the association to early amino acid intake. The results of the instrumental variable analysis confirmed those of the main analysis. Moreover, we did not observe any association between the characteristics of the NICUs and the nutritional strategies used in those NICUs, suggesting a lack of performance bias. The reason a specific nutrition strategy was chosen in a given NICU may have been associated with the medical staff’s interest in nutritional care or the extent of the staff’s agreement with international guidelines. These factors probably explain the wide range of nutritional practices observed in this national cohort, confirming the broad variation in practices observed across Europe.^19^

The second limitation is that the primary outcome was known for only 60% of children in the present study; however, the sensitivity analyses based on the imputed data confirmed the findings of the main analysis. The third limitation is the small number of children with MRI data available, which precludes the performance of correlation mediation analyses to ascertain whether the impact for FSIQ was mediated by the effect on white matter volume or maturation.

## Conclusions

In this nationwide population-based cohort study of preterm infants born at less than 30 weeks’ gestation, high early amino acid intake was independently associated with a greater likelihood of surviving with an FSIQ score greater than −1 SD at age 5 years. Well-designed randomized studies with long-term follow-up and prespecified sex-specific analyses are needed to confirm these results.

## Notes

Supplement 1. **eMethods 1.** Magnetic Resonance Imaging Examination and Interpretation **eMethods 2.** Instrumental Variable Approach **eTable 1.** Characteristics of Preterm Infants Alive at Age 5 Years 6 Months With and Without FSIQ Score **eTable 2.** Association Between Instrumental Variable and Protein Intake of More Than 3.50 g/kg per Day at Day 7 After Birth in the Overall Cohort **eTable 3.** Instrumental Variable and Covariates in the Overall Cohort **eTable 4.** Correlation Between Protein Intake at Day 7 After Birth (as a Continuous Variable) and Data From Magnetic Resonance Imaging at Term in the Matched Cohort **eTable 5.** Correlation Between Protein Intake at Day 7 After Birth (as a Continuous Variable) and Data From Magnetic Resonance Imaging at Term in Overall Cohort **eTable 6.** Nonadjusted Correlation Between FSIQ Assessed at Age 5 Years and Nutritional Data Collected at Days 3 and 7 After Birth in the Overall and Matching Cohorts **eTable 7.** Correlation Between Protein Intake at Day 7 After Birth (as a Continuous Variable) and FSIQ at Age 5 Years in Matching Cohort by Subgroup **eFigure 1.** Association Between NICU Patient Volume and Protein Intake of More than 3.50 g/kg per Day at Day 7 After Birth **eFigure 2.** Propensity Score Distribution in Nonexposed vs Exposed Groups and Receiver Operating Characteristic Curve of the Model **eFigure 3.** Nonadjusted Correlation Between Macronutrient Intake at Days 3, 7, and 28 After Birth and FSIQ in the Matched Cohort **eFigure 4.** Strategies of NICUs According to Quartile of Instrumental Variable, Corresponding to the NICU’s Preference for Protein Intake Greater Than 3.50 g/kg per Day **eFigure 5.** Multivariable Analysis of Survival With FSIQ More Than −1 SD (FSIQ Score ≥93) Among Preterm Infants With Protein Intake Known at Day 7 After Birth, in All Cases Using Multiple Imputations
eReferences

Supplement 2. **Nonauthor Collaborators.** Members of Nutrition EPIPAGE-2 Study Group and EPIRMEX Study Group
