# Supplementary material for: Association Between Early Amino Acid Intake and Full-Scale IQ at Age 5 Years Among Infants Born at Less Than 30 Weeks’ Gestation
Source: JAMA Netw Open. 2021 Nov 30;4(11):e2135452. doi: 10.1001/jamanetworkopen.2021.35452 (PMC8634058; doi:10.1001/jamanetworkopen.2021.35452)
Supplement: Supplement 1. — eMethods 1. Magnetic Resonance Imaging Examination and Interpretation eMethods 2. Instrumental Variable Approach eTable 1. Characteristics of Preterm Infants Alive at Age 5 Years 6 Months With and Without FSIQ Score eTable 2. Association Between Instrumental Variable and Protein Intake of More Than 3.50 g/kg per Day at Day 7 After Birth in the Overall Cohort eTable 3. Instrumental Variable and Covariates in the Overall Cohort eTable 4. Correlation Between Protein Intake at Day 7 After Birth (as a Continuous Variable) and Data From Magnetic Resonance Imaging at Term in the Matched Cohort eTable 5. Correlation Between Protein Intake at Day 7 After Birth (as a Continuous Variable) and Data From Magnetic Resonance Imaging at Term in Overall Cohort eTable 6. Nonadjusted Correlation Between FSIQ Assessed at Age 5 Years and Nutritional Data Collected at Days 3 and 7 After Birth in the Overall and Matching Cohorts eTable 7. Correlation Between Protein Intake at Day 7 After Birth (as a Continuous Variable) and FSIQ at Age 5 Years in Matching Cohort by Subgroup eFigure 1. Association Between NICU Patient Volume and Protein Intake of More than 3.50 g/kg per Day at Day 7 After Birth eFigure 2. Propensity Score Distribution in Nonexposed vs Exposed Groups and Receiver Operating Characteristic Curve of the Model eFigure 3. Nonadjusted Correlation Between Macronutrient Intake at Days 3, 7, and 28 After Birth and FSIQ in the Matched Cohort eFigure 4. Strategies of NICUs According to Quartile of Instrumental Variable, Corresponding to the NICU’s Preference for Protein Intake Greater Than 3.50 g/kg per Day eFigure 5. Multivariable Analysis of Survival With FSIQ More Than −1 SD (FSIQ Score ≥93) Among Preterm Infants With Protein Intake Known at Day 7 After Birth, in All Cases Using Multiple Imputations eReferences [file jamanetwopen-e2135452-s001.pdf]

Supplementary Online Content

Rozé JC, Morel B, Lapillonne A, et al; Nutrition EPIPAGE-2 Study Group; EPIRMEX Study Group. Association between early amino acid intake and full-scale IQ at age 5 years among infants born at less than 30 weeks' gestation. *JAMA Netw Open*. 2021;4(11):e2135452. doi:10.1001/jamanetworkopen.2021.35452

eMethods 1. Magnetic Resonance Imaging Examination and Interpretation

eMethods 2. Instrumental Variable Approach

eTable 1. Characteristics of Preterm Infants Alive at Age 5 Years 6 Months With and Without FSIQ Score

eTable 2. Association Between Instrumental Variable and Protein Intake of More Than 3.50 g/kg per Day at Day 7 After Birth in the Overall Cohort

eTable 3. Instrumental Variable and Covariates in the Overall Cohort

eTable 4. Correlation Between Protein Intake at Day 7 After Birth (as a Continuous Variable) and Data From Magnetic Resonance Imaging at Term in the Matched Cohort

eTable 5. Correlation Between Protein Intake at Day 7 After Birth (as a Continuous Variable) and Data From Magnetic Resonance Imaging at Term in Overall Cohort

eTable 6. Nonadjusted Correlation Between FSIQ Assessed at Age 5 Years and Nutritional Data Collected at Days 3 and 7 After Birth in the Overall and Matching Cohorts

eTable 7. Correlation Between Protein Intake at Day 7 After Birth (as a Continuous Variable) and FSIQ at Age 5 Years in Matching Cohort by Subgroup

eFigure 1. Association Between NICU Patient Volume and Protein Intake of More than 3.50 g/kg per Day at Day 7 After Birth

eFigure 2. Propensity Score Distribution in Nonexposed vs Exposed Groups and Receiver Operating Characteristic Curve of the Model

eFigure 3. Nonadjusted Correlation Between Macronutrient Intake at Days 3, 7, and 28 After Birth and FSIQ in the Matched Cohort

eFigure 4. Strategies of NICUs According to Quartile of Instrumental Variable, Corresponding to the NICU's Preference for Protein Intake Greater Than 3.50 g/kg per Day

eFigure 5. Multivariable Analysis of Survival With FSIQ More Than −1 SD (FSIQ Score ≥93) Among Preterm Infants With Protein Intake Known at Day 7 After Birth, in All Cases Using Multiple Imputations

eReferences

This supplementary material has been provided by the authors to give readers additional information about their work.

© 2021 Rozé JC et al. *JAMA Network Open*.

eMethods 1. Magnetic Resonance Imaging Examination and Interpretation

The children included in this study had an MRI at theoretical term age between 39 and 41 weeks of corrected age, using a 1.5T or 3T MRI system with a dedicated 8-channel head coil, after parental consent. Throughout the scan, infants were monitored using an apnea monitor and an oxygen saturation probe, and if required, oral sucrose was administered. The infants were fed, swaddled and were wearing earplugs

T2 datasets were obtained using axial T2 morphological sequence (fast spin echo/turbospin echo with a 90 flip-back pulse); slice thickness of 3 mm, pixel size 0.39x 0.39 mm, field of view 192 mm, repetition time 6680 ms, echo time 142 ms, and flip angle 120.

An automated brain metrics using a validated automated mathematical approach.[1] with the max-tree representations segmentation method was used by one observer to classify on a single axial T2 section on which the caudate heads, lentiform nuclei, and thalami were maximally visible, as defined in the Kidokoro et al. study.[2] A quality control was performed by a secondary blind observer to determine the most appropriate slice. This slice was segmented into intracranial cavity (after skull stripping), basal ganglia and thalami (BGT), cortical gray matter and white matter.[3] The corresponding surfaces were measured in mm^2.

When it was possible, measurements of anisotropy were performed. Fractional anisotropy (FA) was extracted from diffusion tensor imaging data. We used the following sequences: 3dT1 and DTI. Infants were imaged, either on a 3T Philips or on a 1.5T Siemens scanner, both using an 8-channel coil. On the Philips scanner, structural magnetic resonance image (sMRI) data were acquired with a fast field echo sequence (1.2 mm coronal slices and in-plane resolution of 0.27 mm, repetition time (TR) = 18.9 ms, echo time (TE) = 4.6 ms, flip angle 8°, time of acquisition (Tacq) = 2:34 min) and diffusion magnetic resonance image (dMRI) data were acquired with an echo planar imaging (EPI) sequence (1 b = 0 vol, 32 b = 1000 s/mm^2 volumes, 3 mm axial slices and in-plane resolution of 1.56 mm, TE = 68 ms, TR = 2981 ms, Tacq = 2.35 min). On the Siemens scanner, sMRI data were acquired with a fast low-angle shot (FLASH) sequence (4.0 mm sagittal slices and in-plane resolution of 0.43 mm, TR = 0.38 ms, TE = 5.52 ms, flip angle 90°, Tacq = 2.40 min) and dMRI data were acquired with an EPI sequence (2 b = 0 vol, 24 b = 1000 s/mm^2 volumes, 2.2 mm axial slices and in-plane resolution of 1.97 mm, TE = 98 ms, TR = 7600 ms, Tacq = 2.43 min). Briefly, diffusion-weighted images were corrected for any geometric distortions by using the Diffusion Toolbox in the FSL FMRIB's Software Library (FSL, version 1.4.9, http ://www.fmrib.ox.ac.uk/fsl/). The tensor calculation and tractography were then performed with the Diffusion Toolkit - Trackvis software tools (Diffusion Toolkit, v:0.6.2.2; Trackvis, v:0.6.0.1) with a deterministic FACT algorithm whose stopping criteria are: FA<0.1 and angle>45°. The pathways of our choice are all crucial in development and relatively well-characterized by neuroanatomists: corpus callosum, left and right corticospinal tract, inferior longitudinal, inferior fronto-occipital, superior longitudinal, uncinate fasciculi and the fornix. For the analysis of the specific white matter tracts, regions of interest were manually placed within tracts of interest by using axial and sagittal color FA maps and T2 FLAIR. Mean FA of lesion-free tracts were measured.

© 2021 Rozé JC et al. *JAMA Network Open*.

eMethods 2. Instrumental Variable Approach

We used unit preference for protein intake >3.5 g/kg/d at day 7 as preference-based instrument.[4] We estimated the unit effect in a mixed-effects logistic regression model with protein intake >3.5 g/kg/d at day 7 after birth as dependent variable, maternal and infant characteristics as fixed effects and a random neonatal unit effect. We categorized the estimated random unit effects into quartiles to define our instrumental variable. To assess its validity, we confirmed that the instrument was strongly associated with protein intake >3.5 g/kg/d at day7 (Table S2 in the supplement) and examined the covariate balance across the instrument categories (Table S3 in the supplement). Instrumental variable analysis was carried out using the 2-stage residual inclusion approach,[5] with additional adjustment for gestational age, sex, and birth weight z-score, maternal educational level. The strength of the instrument was formally assessed by the partial F statistic in the first-stage regression model.[6]

© 2021 Rozé JC et al. *JAMA Network Open*.

eTable 1. Characteristics of Preterm Infants Alive at Age 5 Years 6 Months With and Without FSIQ Score

	No./Total (%) or Mean (SD)						
**Alive at 5$^{1/2}$ years**	**Overall Cohort (N =1593/1789)**				**Matched Cohort (N = 1290/1434)**		
**Characteristics**	**Without IQ score N=615**	**With IQ score N=978**	**p**		**Without IQ score N=515**	**With IQ score N=775**	**p**
**Maternal level of education**							
Information missing	81 (13.2%)	31 (3.2%)	<0.001		72 (14.0%)	24(3.1%)	<0.001
less than high school diploma	226 (36.7%)	224 (22.9%)			186 (36.1%)	184 (23.7%)	
High school diploma	115 (18.7%)	202(20.7%)			95 (18.4%)	64 (21.2%)	
Higher than high school diploma	193 (31.4%)	521(53.3%)			162 (31.5%)	403 (52.0%)	
**Gestational age in weeks. mean (SD)**	27.3 (±1.5)	27.3 (±1.4)	0.445		27.3 (1.5)	27.3 (1.5)	0.996
**Male**	323 (52.5%)	498 (50.9%)	0.534		275 (53.4%)	408 (52.6%)	0.791
**Birthweight z-score. mean (SD)**	-0.04 (±0.99)	-0.09 (±1.00)	0.291		-0.02 (±0.97)	-0.09 (±.99)	0.277
**Leading causes of preterm delivery**							
Information missing	19 (3.1%)	24 (2.5%)	0.279		10 (1.9%)	15 (1.9%)	0.380
Twins or triplets	200 (32.5%)	313 (32.0%)			172 (33.4%)	249 (32.1%)	
Singleton with preterm labor	184 (29.9%)	256 (26.2%)			160 (31.1%)	207 (26.7%)	
Singleton with preterm rupture of membranes	93 (15.1%)	159 (16.3%)			74 (14.4%)	123 (15.9%)	
Singleton with vascular disorders without FGR	51 (8.3%)	80 (8.2%)			41 (8.0%)	65 (8.4%)	
Singleton with vascular disorders and FGR	46 (7.5%)	94 (9.6%)			38 (7.4%)	75 (9.7%)	
Singleton with placental abruption	4 (0.7%)	17 (1.7%)			4 (0.8%)	15 (1.9%)	
Singleton with isolated fetal growth retardation	18 (2.9%)	35 (3.6%)			16 (3.1%)	26 (3.4%)	
**Antenatal corticosteroids**							
Information missing	18 (2.9%)	33 (3.4%)	0.719		17 (3.3%)	30 (3.9%)	0.763
No	103(16.7%)	150 (15.3%)			86 (16.7%)	119 (15.4%)	
Incomplete cure	95 (15.4%)	167 (17.1)			80 (15.5%)	133 (17.2%)	
Yes	399 (64.9%)	628 (64.2%)			332 (64.5%)	493 (63.6%)	
**Caesarean section**							

© 2021 Rozé JC et al. *JAMA Network Open*.

Information missing	7 (1.1%)	12 (1.2%)	0.206		3 (0.6%)	6 (0.8%)	0.06
Yes	371 (60.3%)	632 (64.6%)			307 (59.6%)	510 (65.8%)	
**APGAR >7 at 5 minutes after birth**							
Information missing	45 (8.7%)	49 (6.3%)			45 (8.7%)	49 (6.3%)	0.254
Yes	362 (70.3%)	564 (72.8%)			362 (70.3%)	564 (72.8%)	
**Regular intestinal transit during the first week after birth**			6.27				
Information missing	24 (4.7%)	27 (3.5%)			24 (4.7%)	27 (3.5%)	0.563
Yes	279 (54.7%)	422 (54.5%)			279 (54.7%)	422 (54.5%)	
**Acute kidney failure**							
Information missing	11 (2.1%)	19 (2.5%)	3.52		11 (2.1%)	19 (2.5%)	
Yes	35 (6.8%)	45 (5.8%)	21.61		35 (6.8%)	45 (5.8%)	0.727
**Surfactant**							
• Information missing	1 (0.2%)	1 (0.1%)	4.47		1 (0.2%)	1 (0.1%)	
• No	99 (19.2%)	138 (17.9%)	2.49		99 (19.2%)	138 (17.9%)	0.824
• 1 dose	300 (58.3%)	471 (60.8%)	7.53		300 (58.3%)	471 (60.8%)	
• >2 doses	115 (22.3%)	165 (21.3%)	5.9		115 (22.3%)	165 (21.3%)	
**Assisted ventilation at day 7**							
Information missing	0 (0%)	0 (0%)			0 (0%)	0 (0%)	0.563
Yes	198 (38.7%)	311 (40.3)			198 (38.7%)	311 (40.3)	
**Patient volume of NICU where the infant was hospitalized at day 7**							
• <20 infants	76 (14.8%)	129 (16.6%)	25.5		76 (14.8%)	129 (16.6%)	0.660
• 21-30 infants	108 (21.0%)	248 (19.1%)	16.21		108 (21.0%)	248 (19.1%)	
• 31-40 infants	79 (15.3%)	110 (14.2%)	10.73		79 (15.3%)	110 (14.2%)	
• >40 infants	252 (49.9%)	388 (50.1%)	13.15		252 (49.9%)	388 (50.1%)	

© 2021 Rozé JC et al. *JAMA Network Open*.

eTable 2. Association Between Instrumental Variable and Protein Intake of More Than 3.50 g/kg per Day at Day 7 After Birth in the Overall Cohort

Instrumental variable[a]	N (% of total)	Exposed N (row %)[b]	Intelligence quotient at 5 years	
			N (% of the quartile)	mean (SD)
Quartile 1	481 (26.9)	81 (16.8)	262 (54.5)	91.0 (15.3)
Quartile 2	319 (17.8)	103 (32.3)	161 (50.8)	93.3 (15.3)
Quartile 3	533 (29.8)	363 (68.1)	307(58.0)	94.8 (15.9)
Quartile 4	456 (25.5)	391 (85.8)	248 (53.7)	97.2 (15.6)

[a] Instrumental variable is the unit preference
[b] p<0.001

© 2021 Rozé JC et al. *JAMA Network Open*.

eTable 3. Instrumental Variable and Covariates in the Overall Cohort

	No./Total (row %) or Mean (SD)				
**Characteristics**	**Quartile 1 N=481**	**Quartile 2 N=319**	**Quartile 3 N=533**	**Quartile 4 N=456**	**P for trend**
**Maternal level of education**					
Information missing	86 (17.88%)	30 (9.40%)	66 (12.38%)	58 (12.72%)	
less than high school diploma	115 (23.91%)	111 (34.80%)	135 (24.34%)	111 (24.34%)	0.458
High school diploma	77 (16.01%)	67 (21.00%)	96 (18.01%)	92 (20.18%)	
Higher than high school diploma	203 (42.20%)	111 (34.80%)	236 (44.28%)	195 (42.76%)	
**Gestational age**	27.2± (1.5)	27.2 ± 1.5)	27.1 ± 1.5	27.3 (± 1.5)	0.911
Male	257 (53.4%)	152(47.7%)	283 (53.1%)	237 (51.97)	0.980
**Birth weight Zscore**	-0.14 (± 0.96)	-0.07 ± 1.03	-0.10 ± 0.98	-0.04 ± 1.05	0.197
**Leading causes of preterm delivery**					
Information missing	16 (3.33%)	6 (1.88%)	16 (3.00%)	18 (3.95%)	
Twins or triplets	156 (32.43%)	124 (38.87%)	170 (31.89%)	125 (27.41%)	0.065
Singleton with preterm labor	134 (27.86%)	82 (25.71%)	139 (26.08%)	135 (29.61%)	
Singleton with preterm rupture of membranes	73 (15.18%)	32 (10.03%)	98 (18.39%)	88 (19.30%)	
Singleton with vascular disorders and FGR	37 (7.69%)	24 (7.52%)	49 (9.19%)	27 (5.92%)	
Singleton with vascular disorders. without FGR	43 (8.94%)	35 (10.97%)	40 (7.50%)	40 (8.77%)	
Singleton with placental abruption	6 (1.25%)	5 (1.57%)	5 (0.94%)	8 (1.75%)	
Singleton with isolated fetal growth retardation	16 (3.33%)	11 (3.45%)	16 (3.00%)	15 (3.29%)	
**Antenatal corticosteroids**					
Information missing	20 (4.16%)	12 (3.76%)	19 (3.56%)	12 (2.63%)	
No	83 (17.26%)	53 (16.61%)	70 (13.13%)	97 (21.27%)	0.246
Incomplete cure	99 (20.58%)	51 (15.99%)	86 (16.14%)	57 (12.50%)	
Yes	279 (58.00%)	203 (63.64%)	358 (67.17%)	290 (63.60%)	
**Caesarean section**					
Information missing	7 (1.46%)	2 (0.63%)	8 (1.50%)	4 0.88%)	

© 2021 Rozé JC et al. *JAMA Network Open*.

Yes	313 (65.07%)	218 (68.34%)	290 (54.41%)	272 (59.65%)	0.361
**APGAR <7 at 5 minutes after birth**					
Information missing	44 (9.15%)	24 (7.52%)	19 (3.56%)	44 (9.65%)	
Yes	91(18.92%)	75 (23.51%)	114 (21.39%)	108 (23.68%)	0.148
**Regular intestinal transit during the first week after birh**					
Information missing	10 (2.08%)	18 (5.64%)	27 (5.07%)	22 (4.82%)	
Yes	272(56.55%)	157 (49.22%)	269 (50.47%)	231 (50.66%)	0.188
**Acute kidney failure**					
Information missing	11 (2.29%)	16 (5.02%)	13 (2.44%)	14 (3.07%)	
Yes	44 (9.15%)	39 (12.23%)	52 (9.76%)	31 (6.08%)	0.166
**Surfactant**					
Information missing	2 (0.42%)	2 (0.63%)	1 (0.19%)	1 (0.22%)	0.535
No	86 (17.88%)	58 (18.18%)	88 (16.51%)	60 (13.16%)	
1 dose	272 (56.55%)	195 (61.13%)	305 (57.60%)	297 (65.13%)	
2 or 3 doses	121 (25.16%)	64 (20.06%)	137 (25.70%)	98 (21.49%)	
**Respiratory assisted ventilation at day 7**					
Information missing	1 (0.21%)	3 (0.94%)	6 (1.13%)	4 (0.88%)	
Yes	229 (47.21%)	132 (41.38%)	238 (44.65%)	176 (38.60%)	0.023
**Patient volume of NICU where the infant was hospitalized at day 7**					
<20 infants	79 (16.42%)	90 (28.21%)	38 (7.13%)	89 (19.52%)	0.199
21-30 infants	120 (24.95%)	23 (7.21%)	89 (16.70%)	145 (31.80%)	
31-40 infants	37 (7.69%)	104 (32.60%)	37 (6.94%)	65 (14.25%)	
>40 infants	245 (50.94%)	102 (31.97%)	369 (69.23%)	157 (34.43%)	

© 2021 Rozé JC et al. *JAMA Network Open*.

eTable 4. Correlation Between Protein Intake at Day 7 After Birth (as a Continuous Variable) and Data From Magnetic Resonance Imaging at Term in the Matched Cohort

	Matched cohort		
	**n**	**beta (95% confidence interval) ***	**p**
**In 18 NICUs, MRI at term**			
White matter aera (mm^2)	134	144 (3; 285)	0.045
Grey matter (mm^2)	134	-1 (-92; 89)	0.974
Basal ganglia and thalami aera (mm^2)	134	-29 (-67; 9)	0.132
Intracranial aera (mm^2)	134	67 (-73; 208)	0.346
**In 6 NICUs**			
Anisotropy of corpus callosum	50	0.018 (0.016; 0.021)	<0.001
Anisotropy of cortico-spinal tractus right	50	0.022 (0.012; 0.031)	<0.001
Anisotropy of cortico-spinal tractus left	50	0.017 (0.004; 0.029)	0.007
Anisotropy of superior longitudinal fasciculus right	42	0.014 (0.005; 0.024)	0,003
anisotropy of superior longitudinal fasciculus left	42	0.018 (0.010; 0.025)	<0.001

*Adjusted for gestational age, birth weight Z-score, and weighted by the inverse of the propensity score; analysis accounting for clustering on neonatal units.

© 2021 Rozé JC et al. *JAMA Network Open*.

eTable 5. Correlation Between Protein Intake at Day 7 After Birth (as a Continuous Variable) and Data From Magnetic Resonance Imaging at Term in Overall Cohort

	**Overall cohort**		
	**n**	**beta (95% confidence interval) ***	**p**
**In 18 NICUs, MRI at term**			
White matter aera (mm^2)	170	170 (30; 310)	0.017
Grey matter (mm^2)	170	-2 (-93; 88)	0.962
Basal ganglia and thalami aera (mm^2)	170	-19 (-57; 19)	0.327
Intracranial aera (mm^2)	170	80 (-57; 216)	0.255
**In 6 NICUs**			
Anisotropy of corpus callosum	62	0.018 (0.015; 0.021)	<0.001
Anisotropy of cortico-spinal tractus right	62	0.020 (0.011; 0.030)	<0.001
Anisotropy of cortico-spinal tractus left	62	0.014 (-0.001; 0.029)	0.064
Anisotropy of superior longitudinal fasciculus right	51	0.012 (0.005; 0.019)	0,002
anisotropy of superior longitudinal fasciculus left	51	0.017 (0.010; 0.025)	<0.001

*Adjusted for gestational age, birth weight Z-score, and weighted by the inverse of the propensity score; analysis accounting for clustering on neonatal units.

© 2021 Rozé JC et al. *JAMA Network Open*.

eTable 6. Nonadjusted Correlation Between FSIQ Assessed at Age 5 Years and Nutritional Data Collected at Days 3 and 7 After Birth in the Overall and Matching Cohorts

	**Overall cohort**		**Matching cohort**	
	**r**	**p**	**r**	**p**
**Day3**				
N	890		674	
Protids	**0.120**	**<0.001**	**0.099**	**0.010**
Carbohydrates	-0.009	0.785	-0.018	0.644
Lipids	0.056	0.095	0.049	0.200
Energy	0.050	0.136	0.037	0.338
Protein/Energy	**0.095**	**0.005**	**0.078**	**0.042**
**Day7**				
N	928		701	
Protids	**0.134**	**<0.001**	**0.106**	**0.004**
Carbohydrates	0.023	0.928	0.025	0.513
Lipids	0.019	0.573	0.010	0.799
Energy	0.047	0.154	0.038	0.309
Protein/Energy	**0.082**	**0.012**	0.066	0.079

© 2021 Rozé JC et al. *JAMA Network Open*.

eTable 7. Correlation Between Protein Intake at Day 7 After Birth (as a Continuous Variable) and FSIQ at Age 5 years in Matching Cohort by Subgroup

	**Non Exposed** n with FSIQ /N	**Exposed** n with FSIQ /N	Total n with FSIQ	**beta (95% confidence interval)***	**p**
**Maternal level of education**					
high school diploma or less	181/426	191/422	372	1.65 (-0.88; 4.17)	0.201
Higher than high school diploma or not known	198/291	205/295	403	3.32 (0.91; 1.23)	0.007
**Early severe illness**					
Yes	115/241	111/224	226	4.05 (0.98; 7.12)	0.010
No	264/476	285/493	559	1.62 (-0.56; 3.8)	0.145
**Late onset infection(s)**					
Yes	211/386	211/377	422	3.04 (0.51; 5.56)	0.018
No	153/300	166/307	319	1.57 (-1.22; 3.50)	0.334
**Morbidities**					
Preterm without BPD	304 /500	296/493	600	2.09 (0.19; 3.98)	0.031
Preterm with BPD	70/131	90/147	160	3.31 (-0.7; 7.33)	0.106
Preterm without NEC	360/669	374/676	734	2.58 (0.83; 4.34)	0.004
Preterm with NEC	17/42	15/31	32	1.46 (-11.43; 14.36)	0.824
Preterm without severe cerebral lesions	360/628	365/635	725	2.47 (0.65; 4.30)	0.008
Preterm with severe cerebral lesions	19/87	25/72	44	1.54 (-4.17; 7.26)	0.597
**Brestfeeding at discharge**					
Yes	140/212	150/223	290	1.82 (-0.38; 4.01)	0.105
No or not known	239/505	246 /494	485	2.68 (0.33; 5.00)	0.025

* analysis accounting for clustering on neonatal units.

© 2021 Rozé JC et al. *JAMA Network Open*.

eFigure 1. Association Between NICU Patient Volume and Protein Intake of More than 3.50 g/kg per Day at Day 7 After Birth

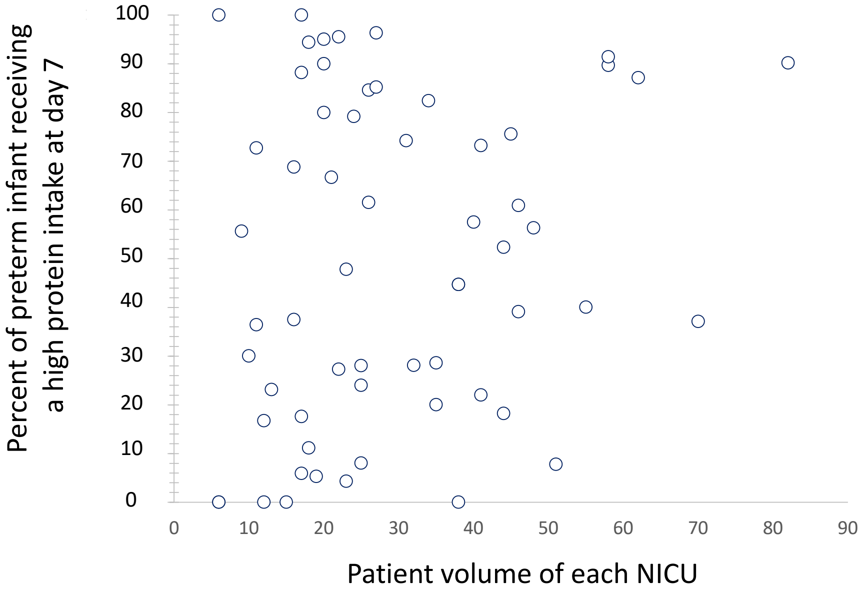

© 2021 Rozé JC et al. *JAMA Network Open*.

eFigure 2. Propensity Score Distribution in Nonexposed vs Exposed Groups and Receiver Operating Characteristic Curve of the Model

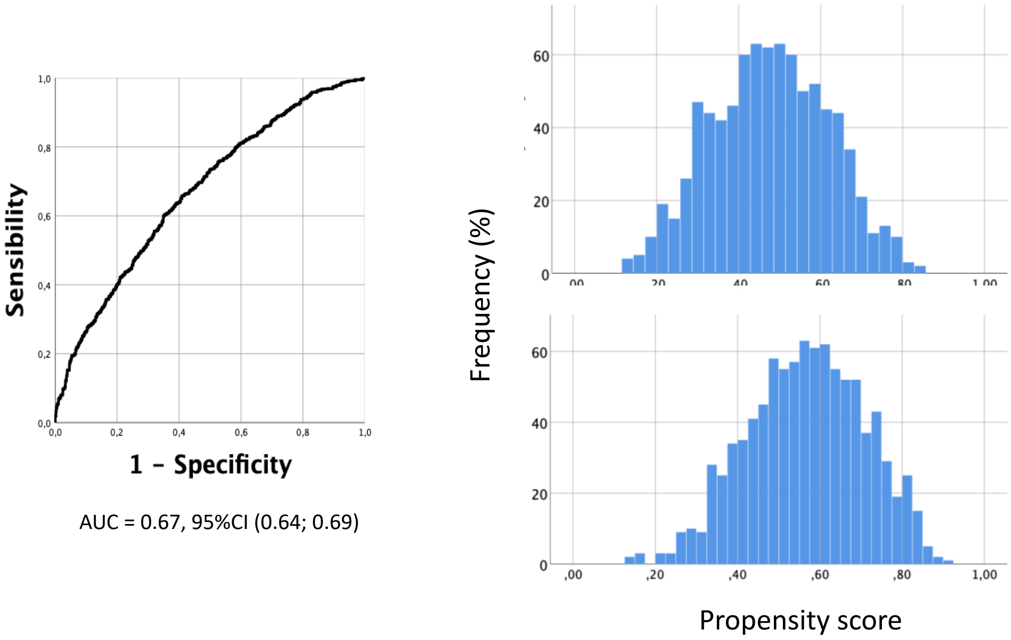

© 2021 Rozé JC et al. *JAMA Network Open*.

eFigure 3. Nonadjusted Correlation Between Macronutrient Intake at Days 3, 7, and 28 After Birth and FSIQ in the Matched Cohort

Day 3	% of parenteral Day3	Amino-acid Day3	Carbohydrate Day3	Lipid Day3	FSIQ
% of parenteral Day3	1				
Amino-acid Day3	.057*	1			
Carbohydrate Day3	-0 .020	.283**	1		
Lipid Day3	- .362**	.379**	.229**	1	
FSIQ	-0 .008	.093*	-0 .020	0 .039	1

Day 7	% of parenteral Day7	Amino-acid Day7	Carbohydrate Day7	Lipid Day7	FSIQ
% of parenteral Day7	1				
Amino-acid Day7	.330**	1			
Carbohydrate Day7	.125**	.329**	1		
Lipid Day7	- .393**	.137**	.173**	1	
FSIQ	-0 .034	.106**	-0 .027	-0 .013	1

Day 3 . Day 7 and Day28	Amino acid Day7	Amino acid Day3	Amino acid Day28	Glucid Day3	Lipid Day3	Glucid Day7	Lipid Day28	Glucid Day28	Lipid Day28	FSIQ
Amino acid Day7	1									
Amino acid Day3	.383**	1								
Amino acid Day28	.093**	0 .028	1							
Glucid Day3	.128**	.283**	-0 .001	1						
Lipid Day3	.069**	.379**	0 .024	.229**	1					
Glucid Day7	.329**	.080**	0 .007	.578**	0 .044	1				
Lipid Day28	.137**	.078**	-0 .005	.126**	.394**	.173**	1			
Glucid Day28	-0 .046	- .094**	.274**	.216**	-0 .051	.213**	- .060*	1		
Lipid Day28	-0 .008	0 .011	0 .029	0 .038	.209**	0 .052	.262**	-0 .002	1	
FSIQ	.106**	.093*	0 .019	-0 .020	0 .039	-0 .027	-0 .013	-0 .056	-0 .008	1

r de spearman

- < -0.30
- ≤ -0.06
- ≥ -0.06
- >0.10
- >0.20
- >0.30

*P<0.05 , ** P<0.01, FISQ : Full-scale intelligent quotient.

© 2021 Rozé JC et al. *JAMA Network Open*.

eFigure 4. Strategies of NICUs According to Quartile of Instrumental Variable, Corresponding to the NICU's Preference for Protein Intake Greater Than 3.50 g/kg per Day

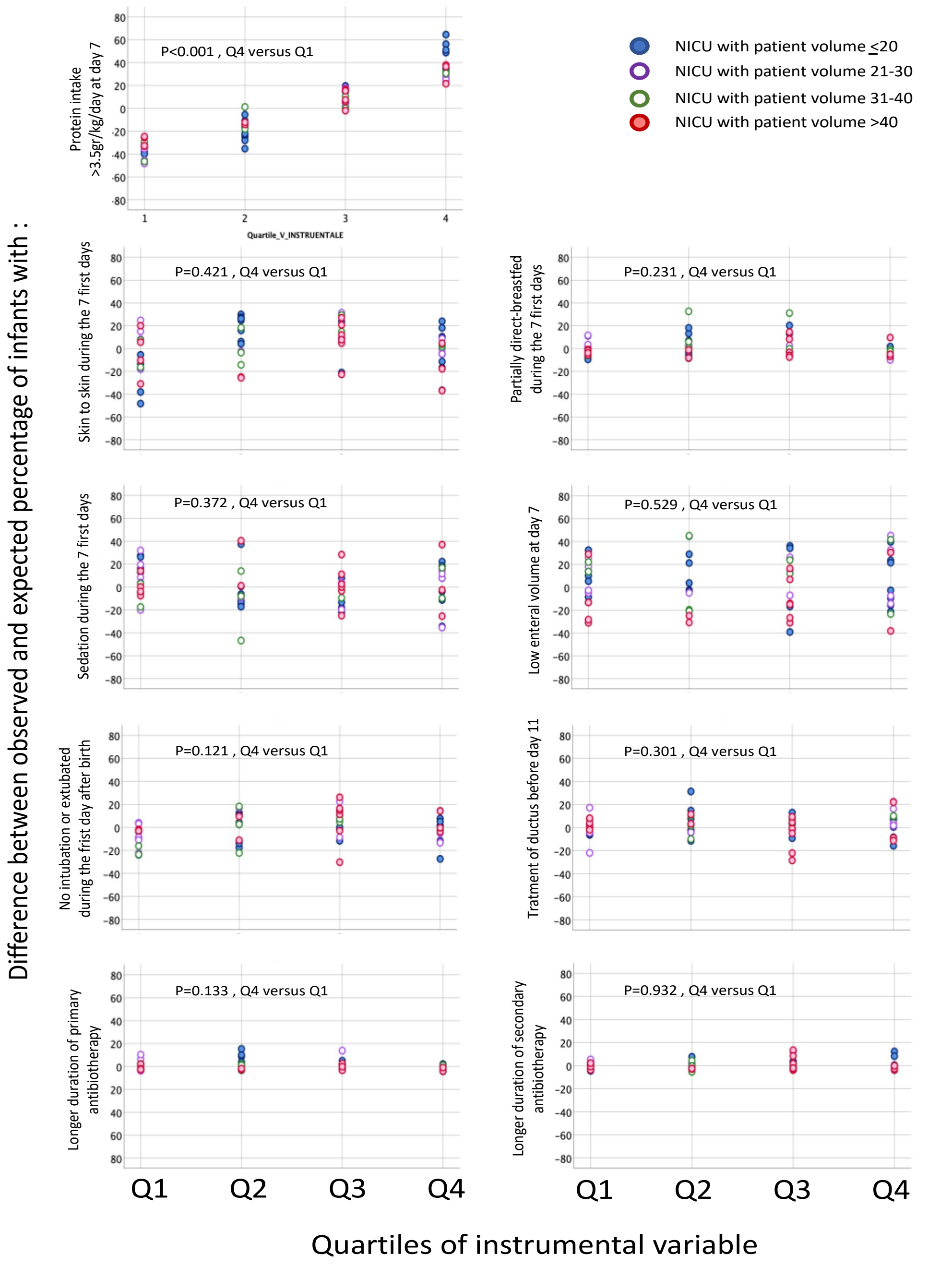

Each circle represents one neonatal intensive care units NICU. We characterized 8 other strategies for each NICU as previously described [7] : intubation or extubation during the first 24 hours after birth, use of sedation during the first week after birth, direct breastfeeding during the first week after birth, skin to skin during the first week after birth, and treatment of ductus arteriosus during the first ten days after birth, speed of progression of enteral feeding during the first week after birth, duration of primary antibiotherapy started during the first 48 hours after birth and duration of secondary antibiotherapy. For each strategy and each NICU, an expected percentage of infants exposed to the strategy has been calculated. On Y axis, we have represented the observed percentage minus the expected percentage.

© 2021 Rozé JC et al. *JAMA Network Open*.

eFigure 5. Multivariable Analysis of Survival With FSIQ More Than −1 SD (FSIQ Score ≥93) Among Preterm Infants With Protein Intake Known at Day 7 After Birth, in All Cases Using Multiple Imputations

Multiple imputations

Part A. No of children with full-scale intelligence quotient at 5 year ≥ -1 standard deviation/ No of Patients (%)

Methods	Non-Exposed	Exposed	Odds Ratio (95% confidence interval)	p
	342*/644 (53.1%)	391*/646 (60.5%)	1.353 (1.047-1.749)	0.021
Overall cohort				
• Model 1	380*/735 (51.6%)	520*/858 (60.7%)	1.374 (1.082 – 1.744)	0.003
• Model 2	380*/735 (51.6%)	520*/858 (60.7%)	1.531 (1.257 – 1.865)	<0.001
• Model 3	380*/735 (51.6%)	520*/858 (60.7%)	1.531 (1.154 – 2.032)	<0.001

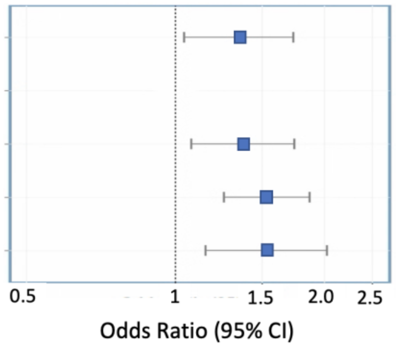

Part B. Coefficient between protein intake at day 7 and full-scall intelligence quotient at 5 year

Methods	n	Beta (95% confidence interval)	p
Propensity-matched cohort	1290	3.00 (0.65 – 5.35)	0.027
Overall cohort			
• Model 1	1593	2.93 (0.83 – 5.02)	0.003
• Model 2	1593	4.44 (2.10 – 6.78)	<0.001
• Model 3	1593	4.44 (1.58 – 7.30)	<0.001

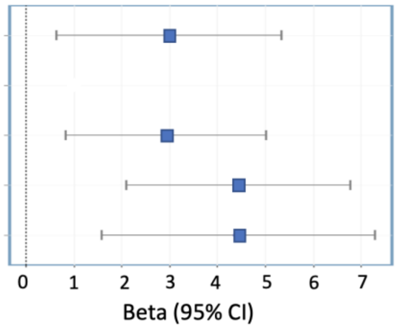

In part A of the figure, the position of each square represents the point estimate of the exposition effect. Horizontal lines represent 95% confidence intervals. Results are expressed as number of event / number of patients. In part B, the position of each square represents the point estimate of the beta coefficient between protein intake at day 7 as continuous variable and full-scale intelligence quotient at $5^{1/2}$ year.

- Model 1, adjusted for gestational age, sex, birthweight z-score and mother's level education.
- Model 2, adjusted for gestational age and weighted by the inverse of the propensity score.
- Model 3, adjusted for gestational age and weighted by the inverse of the propensity score; analysis accounting for clustering on neonatal units.

© 2021 Rozé JC et al. *JAMA Network Open*.

eReferences

1. Morel B, Bertault P, Favrais G, et al. Automated brain MRI metrics in the EPIRMEX cohort of preterm newborns: Correlation with the neurodevelopmental outcome at 2 years. Diagn Interv Imaging 2021;102:225-32.
2. Kidokoro H, Neil JJ, Inder TE. New MR Imaging Assessment Tool to Define Brain Abnormalities in Very Preterm Infants at Term. Am J Neuroradiol 2013;34:2208–14
3. Xu Y, Morel B, Dahdouh S, et al. The challenge of cerebral magnetic resonance imaging in neonates: A new method using mathematical morphology for the segmentation of structures including diffuse excessive high signal intensities. Med Image Anal 2018;48:75–94.
4. Stukel TA, Fisher ES, Wennberg DE, et al. Analysis of observational studies in the presence of treatment selection bias: effects of invasive cardiac management on AMI survival using propensity score and instrumental variable methods. JAMA. 2007;297(3):278-285.
5. Greenland S. An introduction to instrumental variables for epidemiologists. International Journal of Epidemiology. 2000; 29(4):722-729.
6. Terza JV, Basu A, Rathouz PJ. Two-stage residual inclusion estimation: addressing endogeneity in health econometric modeling. J Health Econ. 2008; 27(3):531-543.
7. Rozé JC, Ancel PY, Marchand-Martin L, et al. Assessment of Neonatal Intensive Care Unit Practices and Preterm Newborn Gut Microbiota and 2-Year Neurodevelopmental Outcomes. JAMA Netw Open 2020;3:e2018119.

© 2021 Rozé JC et al. *JAMA Network Open*.
