# Supplementary material for: Association Between Early Amino Acid Intake and Full-Scale IQ at Age 5 Years Among Infants Born at Less Than 30 Weeks’ Gestation
Source: JAMA Netw Open. 2021 Nov 30;4(11):e2135452. doi: 10.1001/jamanetworkopen.2021.35452 (PMC8634058; doi:10.1001/jamanetworkopen.2021.35452)
Supplement: Supplement 2. — Nonauthor Collaborators. Members of Nutrition EPIPAGE-2 Study Group and EPIRMEX Study Group [file jamanetwopen-e2135452-s002.pdf]

Supplemental Online Content: Nonauthor Collaborators

*Indicates required information. Only first name, last name, and suffix will appear in PubMed.

*Group Name(s): Nutrition EPIPAGE-2 Study Group and EPIRMEX Study Group							
***First Name and Middle Initial(s)**	***Last Name**	***Suffix (eg, Jr, III)**	Academic Degrees	Institution	Location (city, state/province, country)	Role or Contribution, eg, chair, principal investigator	Group (if more than 1 Group listed in the byline) and/or Subgroup (eg, Steering Committee)
Farid	Bourdred		MD, PhD	Department of neonatalogy, APHM	Marseille, France	Acquisition of data	Nutrition EPIPAGE 2 study group
Odile	Dicky		MD	Department of neonatal medicine, Toulouse unisversity hospital	Toulouse, France	Acquisition of data	Nutrition EPIPAGE 2 study group
Jean-Michel	Hascoet		MD,PhD	Department of neonatal medicine, Nancy unisversity hospital	Nancy, France	Acquisition of data	Nutrition EPIPAGE 2 study group
Gerard	Thiriez		MD,PhD	Department of neonatal medicine,Besancon unisversity hospital	Besancon, France	Acquisition of data	Nutrition EPIPAGE 2 study group
Luc	Desfrere		MD,PhD	Department of neonatal medicine, Louis Mourier Hospital	Colombes, France	Acquisition of data	Nutrition EPIPAGE 2 study group
Clement	Chollat		MD,PhD	Department of neonatal medicine, Cochin university hospital	Paris, France	Acquisition of data	Nutrition EPIPAGE 2 study group
Isabelle	Filipiak		MD, PhD	INSERM UMR930, Tours university	Tours, France	Acquisition and interpretation of data	EPIRMEX study group
Dominique	Sirinelli		MD, PhD	INSERM UMR1253, Tours university	Tours, France	Acquisition and interpretation of data	EPIRMEX study group
Alexandre	Chadi		MD	Department of neonatal medicine, Rouen university hospital	Rouen, France	Acquisition and interpretation of data	EPIRMEX study group
Catherine	Adamsbaum		MD,PhD	Pediatric Radiology Department, Kremlin Bicetre university hospital	Kremlin Bicetre	Acquisition and interpretation of data	EPIRMEX study group
